# SILAC analysis of *Escherichia coli* proteome during progression of growth from exponential to prolonged stationary phase

**DOI:** 10.1128/mra.00042-24

**Published:** 2024-04-23

**Authors:** Kaspar Reier, Aivar Liiv, Jaanus Remme

**Affiliations:** 1 Institute of Molecular and Cell Biology, University of Tartu, Tartu, Estonia; University of Guelph, Guelph, Ontario, Canada

**Keywords:** *Escherichia coli*, stationary phase, proteome, SILAC

## Abstract

The expression level of individual proteins varies markedly during the progression of the growth phase in bacteria. A set of proteins was quantified in *Escherichia coli* total proteome during 14 days of batch cultivation using pulse stable isotope labeled amino acids in cell culture (SILAC)-based quantitative mass spectrometry.

## ANNOUNCEMENT

**Fig 1:**
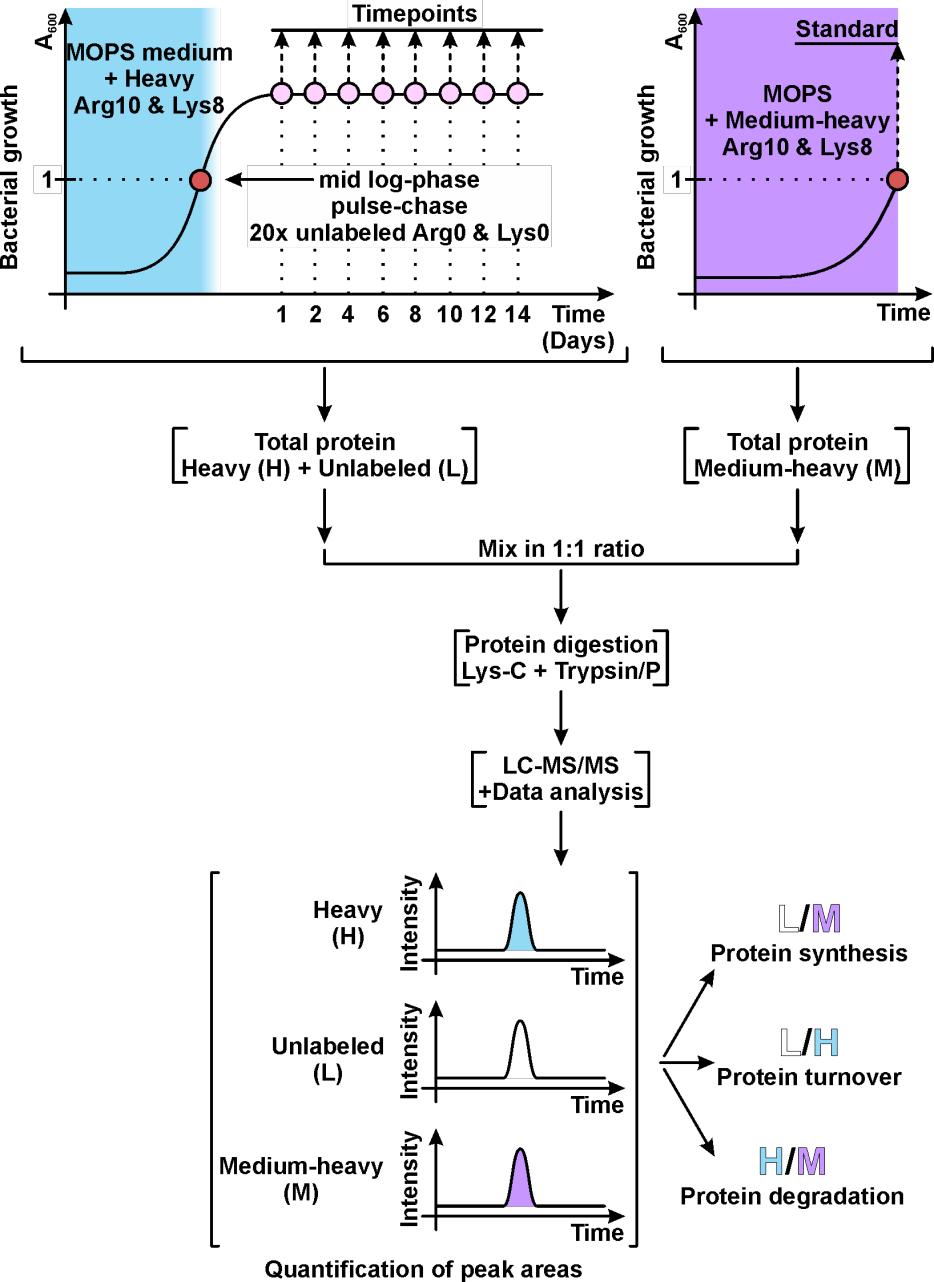
Experimental workflow for SILAC-based proteomic analysis of *Escherichia coli* during growth from exponential to prolonged stationary phase.

This study aimed to determine protein quantities in *Escherichia coli* proteome. The novelty of this work lies in time. Previously, *Escherichia coli* proteome has been quantified in the early stationary phase (1). However, this work also quantified the proteome during the progression into a prolonged stationary phase. The stable isotope labeled amino acids in cell culture (SILAC)-based experimental approach was used (Fig. 1) (2). Three biological replicates of the *Escherichia coli* MG1655-SILAC *(F−*, λ*−, rph-1*, Δ*lysA*, and Δ*argA*) (3) strain were grown at 37°C in the MOPS medium (4), containing glucose (final concentration 2 mg/mL), thiamine (0.1 µg/mL), and amino acids (0.1 mg/mL). Heavy (H) labeled arginine and lysine were used (Arg10: [^13^C]_6_H_14_[^15^N]_4_O_2_) and (Lys8: [^13^C]_6_H_14_[^15^N]_2_O_2_) (Silantes, Germany). At the mid-log phase (*A*_600_ ≈ 1.0), the cultures were pulsed with a 20-fold molar excess (final concentration: 2.0 mg/mL) of unlabeled (L) arginine (Arg0: C_6_H_14_N_4_O_2_) and lysine (Lys0: C_6_H_14_N_2_O_2_) (Sigma, Germany). The cultures were divided into eight aliquots and incubated at 37°C. Cells were collected on day 1 (24 h after the start of the culture), day 2 (48 h), and subsequently in 48-h intervals over the following 12 days. For the standard, the same strain was grown in the MOPS medium containing medium-heavy (M) labeled arginine (Arg6: [^13^C]_6_H_14_N_4_O_2_) and lysine (Lys4: C_6_H_10_[^2^H]_4_N_2_O_2_) (Silantes, Germany). Standard cells were collected in the mid-log phase (*A*_600_ ≈ 1.0).

Cells were lysed by suspension in 10 volumes of lysis buffer (4% SDS, 100 mM Tris‐HCl [pH 7.5], and 100 mM dithiothreitol [DTT]) and consequent heating at 95°C for 5 min. Samples were homogenized by sonication (Bandelin, Germany) (60 × 1 s pulses at 50% intensity). Cell debris was removed by centrifugation at 14,000 × *g* for 10 min. Protein concentration in lysates was determined with a Micro BCA Protein Assay Kit (Thermo Fisher Scientific, Germany). Total protein from each timepoint, containing H and L arginine and lysine, was mixed with total protein from standard, containing M labeled arginine and lysine, in a 1:1 ratio (Fig. 1). This provides an internal reference and separates protein synthesis and degradation rate measurements. The decreasing ratio of H/M isotopes measures the rate of protein degradation. In contrast, the increasing ratio of L/M indicates protein synthesis, and the change in the L/H ratio measures the rate of net protein turnover.

Proteins were precipitated with 2:1:3 vol methanol:chloroform:water. Protein pellets were suspended in 25 µL of 7 M urea and 2 M thiourea, followed by disulfide reduction with 5 mM DTT for 30 min and cysteine alkylation with 10 mM chloroacetamide for 30 min at room temperature. Proteins were digested with endoproteinase Lys-C (Wako, Germany) at a 1:50 enzyme-to-protein ratio for 4 h. Urea concentration in the solution was reduced by adding four volumes of 100 mM ABC. Peptides were digested overnight using mass spectrometry-grade trypsin (enzyme-to-protein ratio 1:50) (Sigma Aldrich, Germany). Enzymes were inactivated with trifluoroacetic acid (final concentration: 1%). Peptides were desalted with self-made reverse-phase C_18_ StageTips columns (5).

Samples were injected into an UltiMate 3000 RSLCnano system using a C_18_ trap‐column (Dionex) and an in‐house packed (3 µm C_18_ particles, Dr Maisch) analytical 50 cm × 75 µm emitter‐column (New Objective, USA). Peptides were eluted at 200 nL/min with a 5%–35% B 240 min gradient (buffer B: 80% acetonitrile + 0.1% formic acid, buffer A: 0.1% formic acid) to a Q Exactive Plus mass spectrometer (MS) (Thermo Fisher, Germany), using a top 10 data‐dependent acquisition strategy. Briefly, one 350–1,400 *m*/*z* MS scan at a resolution setting of *R* = 70,000 at 200 *m*/*z* was followed by higher‐energy collisional dissociation fragmentation (normalized collision energy of 26) of 10 most intense ions (*z*: +2 to +6) at *R* = 17,500. MS and MS/MS ion target values were 3*e*6 and 5*e*4 with 50 ms injection times. Dynamic exclusion was limited to 70 s.

Previously, we used this data set to determine stability, degradation, and synthesis rates for ribosomal proteins (2) and ribosome-associated proteins (6). Under the conditions presented in these studies, approximately 2,000 proteins were identified in the data set over all timepoints (2, 6).

## Data Availability

Mass spectrometry data are archived in ProteomeXchange via the PRIDE database, Project PXD035927.
